# Q Fever in Military Firefighters during Cadet Training in Brazil

**DOI:** 10.4269/ajtmh.17-0979

**Published:** 2018-06-25

**Authors:** Elba Regina Sampaio de Lemos, Tatiana Rozental, Bibiana Nogueira Siqueira, Adonai Alvino Pessoa Júnior, Thays Euzebio Joaquim, Raphael Gomes da Silva, Carolina de Andrade Leite, Adriana Alvarez Arantes, Marisângela Ferreira da Cunha, Danielle Provençano Borghi

**Affiliations:** 1 Laboratório de Hantaviroses e Rickettsioses, Instituto Oswaldo Cruz, Fiocruz, Rio de Janeiro, Brazil;; 2 Hospital Central Aristarcho Pessoa–Corpo de Bombeiros Militar do Estado do Rio de Janeiro (CBMERJ), Rio de Janeiro, Brazil;; 3 Hospital São Francisco de Assis, Rio de Janeiro, Brazil;; 4 Hospital Universitário Pedro Ernesto, Universidade do Estado do Rio de Janeiro (UERJ), Rio de Janeiro, Brazil;; 5 Hospital Municipal Jesus, Rio de Janeiro, Brazil;; 6 Laboratório Rocha e Fonseca, Rio de Janeiro, Brazil

## Abstract

We report five cases of Q fever among cadets during a training program for Military Firefighters Academy in the state of Rio de Janeiro, Brazil. This cluster confirms the significance of *Coxiella burnetii* as an infectious agent in Brazil, where the occurrence of this zoonosis is poorly documented and highlights the potential risk for Q fever transmission in rural areas or farms with infected animals.

Q fever is a zoonosis caused by *Coxiella burnetii*, an intracellular bacterium that infects several animals, including humans. After an incubation period of 14–37 days, most human infections are asymptomatic but can present as a flu-like illness, pneumonia, or hepatitis.^1^ Hepatitis and endocarditis or fatal cases depend on the patient’s risk factors and the virulence of the bacterial strain.^1,2^ The infection is transmitted to humans from infected animals through contact with and inhalation of secretions, particularly birth products from domestic ruminants.^1^

*Coxiella burnetii* was first reported in Australia in 1937 and has emerged in several regions of the world in the last decade.^2,3^ Thus, Q fever has become an important public health concern in the northern hemisphere, particularly in European countries, where cases have been reported in areas where Q fever was previously nonendemic, such as the Netherlands.^4^ Outbreaks and sporadic human cases are reported around the world, but there is little knowledge about Q fever in Africa and South America.^5^ In Brazil, the first serological evidence of *C. burnetti* infection was described in 1953. However, only recently, a few rare cases have been identified in the southeast region.^5–8^ Curiously, an annual incidence of 150 cases/100,000 people was observed in 2005 in French Guiana, which borders the northern region of Brazil.^5^ Thus, this disparity with Brazil suggests that the occurrence of Q fever may be underreported.

We describe a cluster of Q fever cases among cadets enrolled in a training program at the Pedro II Military Firefighters Academy (MFAPRJ) in Rio de Janeiro, Brazil.

In October 2016, five cadets with acute febrile illness were evaluated at the Central Hospital of MFAPRJ (Table 1). All patients were male, aged between 20 and 25 years, and had a history of camping during military survival training, with more than 42 cadets (total of 47) in the region of Ribeirão das Lajes (22°41′28″S 43°51′49″W) in the state of Rio de Janeiro from September 29 to October 13, 2016. They reported numerous tick bites, and 45 cadets participated directly in slaughtering animals for their own consumption (including a goat) under field survival conditions.

**Table 1:** Clinical and laboratorial findings of Q fever in cadets in Brazil, 2016

Case (age)		Date of illness onset	Clinical manifestations	Incubation period	Hemogram	Thorax and abdominal computed tomography	IFA (serum samples–phase II IgG) (date of sample collection)					PCR		Comments
							1S	2S	3S	4S	5S	Blood	BAL	
1 (20)		October 17	Fever (38°C), dry cough, myalgia, headache, and abdominal pain	18	142,000 leukocytes	Consolidation in the left upper lobe, lingula, and right lower lobe with air bronchogram. Pleural effusion on the left side. Hepatosplenomegaly	< 64 (October 27)	128 (November 01)	1,024 (November 07)	2,048 (November 18)	16,384 (November 30)	NEG	NA	Nonhospitalized patient
					467,000 platelets									
2 (22)		October 21	Fever (38.4°C), dysuria, myalgia, dry cough, fatigue, headache, and dyspnea	22	7,700 leukocytes	Consolidation in the left lower lobe, with infiltrates “frosted glass” associated with air bronchogram. Hepatosplenomegaly	< 64 (October 27)	512 (November 01)	4,096 (November 07)	4,096 (November 16)	16,384 (November 30)	NEG	NA	Hospitalized patient
					178,000 platelets									
3 (23)		October 23	Fever (38°C), myalgia, dry cough, and headache	31	10,400 leukocytes	Consolidation in the right lung with air bronchogram.	< 64 (October 27)	128 (November 01)	1,024 (November 07)	256 (November 18)	128 (November 30)	NEG	NA	Hospitalized patient
					194,000 platelets	Splenomegaly								
4 (21)		October 23	Fever (39°C), dry cough, headache, and acute respiratory failure	24	8,700 leukocytes	Consolidation in the left lung.	< 64 (October 27)	128 (November 01)	1,024 (November 07)	2,048 (November 18)	16,384 (November 30)	NEG	POS	Patient hospitalized in intensive care
					227,000 platelets	Hepatosplenomegaly								
5 (22)		October 29	Fever (38.1°C), nausea, vomit, abdominal pain, myalgia, dry cough, and headache	30	7,900 leukocytes	Consolidation in the left lung.	< 64 (November 11)	128 (November 16)	1,024 (November 30)	NA	NA	NEG	NA	Hospitalized patient
					193,000 platelets	Hepatosplenomegaly								

NA = not available; NEG = negative; IFA = indirect immunofluorescence test; PCR = polymerase chain reaction; POS = positive; S = sample. Normal ranges: leukocytes 4,000–11,000; platelets 150,000–450,000.

One of the cadets had severe pneumonia associated with respiratory failure. Laboratory findings and a chest radiograph were available from medical records and are reported in the table. Because of the possibility of diseases associated with ticks, 200 mg/day of doxycycline were administered after collecting blood samples from the patients and bronchoalveolar lavage (BAL) from the one patient who progressed to respiratory failure. All patients recovered and were discharged.

An indirect immunofluorescence assay (IFA) (Focus Diagnostics^™^, Cypress, CA) for antibodies against *C. burnetii* was performed with a titer cutoff of 64. Sequential serum samples were collected from the patients, and in all cases, paired serum samples showed a > 4-fold increase of immunoglobulin G (phases II) specific to *C. burnetii*. Blood and BAL samples were analyzed using polymerase chain reaction (PCR) for a fragment of IS*1111* gene—a transposon-like repetitive region, as reported previously.^7,8^ *Coxiella burnetii* DNA was detected in BAL from patient 4, and the sequence fragment revealed 99% sequence identity with the homologous gene fragment of the *htp*AB transposase gene from *C. burnetii* strains RSA 331, RSA 439, and RSA 443 (GenBank accession no. MF447442).

In December 2016, serum samples from the 42 healthy military cadets who participated in the training program were analyzed, and all were nonreactive. Considering that the probable source of infection was the goat, a field investigation was also carried out on a commercial shop where the cadets purchased the animal. Blood and anal swab samples collected from 12 goats were analyzed using IFA and PCR. The results revealed an absence of *C. burnetii* infection. Unfortunately, given the nature of most small ruminant markets in Brazil, it was not possible to identify the rural property that supplied the goat to the commercial place.

Previous studies have identified *C. burnetii* as one of the pathogens for outbreaks of influenza-like illness and pneumonia among military personnel. Military personnel are a high-risk occupational group that is often exposed to several infectious agents.^9–11^ This is the first report of a Q fever outbreak among military personnel in Brazil, where autochthonous cases have mainly been reported in the states of Rio de Janeiro and São Paulo.^6–8^

All five patients in this study had contact with the goat, and their estimated incubation periods ranged from 18 to 31 days. The estimated attack rate was 10.6% (5/47). However, cases 2 and 4 reported close contact with the ruminant and had more severe disease courses than the other patients. This confirms that the clinical severity also depends on the size of the inoculum. Outbreaks are being identified more frequently among military personnel around the world, and an increasing number of cases are being reported in Latin America. Together with the present data, these facts emphasize the need for further work to generate additional data on *C. burnetii* as an infectious agent in the general population and the significance of Q fever in the military in Brazil.
